# 3D-Printed Oral Disintegrating Films of Brain-Targeted Acetyl Salicylic Acid Nanoparticles for Enhanced CNS Delivery in Ischemic Stroke

**DOI:** 10.3390/pharmaceutics17121547

**Published:** 2025-11-30

**Authors:** Dedeepya Pasupuleti, Marissa D’Souza, Amarae Ferguson, Mahek Anil Gulani, Parth Patel, Revanth Singh, Emmanuel Adediran, Sharon Vijayanand, Tanisha Manoj Arte, Martin D’Souza

**Affiliations:** 1 Department of Pharmaceutical Sciences, Larkin College of Pharmacy, 18301 North Miami Ave, Miami, FL 33169, USA; dpasupuleti@larkin.edu (D.P.); emmanuel.adediran@live.mercer.edu (E.A.); 2 Center for Nanosphere Technology, Department of Pharmaceutical Sciences, College of Pharmacy, Mercer University, Atlanta, GA 30341, USA; amarae.ferguson@live.mercer.edu (A.F.); mahekanil.gulani@live.mercer.edu (M.A.G.); parthupatel242@gmail.com (P.P.); revanth.singh.sateesh@live.mercer.edu (R.S.); sharon.c.p.vijayanand@live.mercer.edu (S.V.); tanisha.manoj.arte@live.mercer.edu (T.M.A.); 3 Vascular Neurology, University of California Medical Center, San Diego, CA 92103, USA; marissadsouza2021@gmail.com

**Keywords:** acetyl salicylic acid, aspirin, ASA, neuroprotective, ischemic stroke, non-invasive route of administration, buccal delivery, 3-D printing, mucoadhesive films, brain targeting, nanotechnology

## Abstract

**Background/Objectives**: Oral administration remains the most widely used route for drug delivery but is unsuitable for many central nervous system (CNS) therapeutics due to extensive hepatic first-pass metabolism and the restrictive blood–brain barrier (BBB). Acetyl salicylic acid (ASA), despite its neuroprotective and anti-inflammatory potential, exhibits poor brain bioavailability when delivered orally, limiting its therapeutic utility in ischemic stroke and chronic neurodegenerative conditions. **Methods**: This study reports the first use of three-dimensional (3D) bioprinting to develop brain-targeting ASA nanoparticle (NP)-loaded orally disintegrating films (ODFs) for direct systemic uptake and enhanced CNS delivery. The ODFs were fabricated using a CELLINK INKREDIBLE plus^®^ bioprinter and optimized for uniformity, rapid dissolution, and nanoparticle stability. **Results:** The films displayed consistent physicochemical properties (weight 10.86 ± 0.28 mg; thickness 0.47 ± 0.26 mm; pH 7.5–7.7) and disintegrated within 2.38 ± 0.28 min. In vitro testing on BEND3 brain endothelial cells confirmed biocompatibility, with no inflammatory response or cytotoxicity up to 62 µg/mL. In vivo biodistribution in murine models demonstrated substantial brain accumulation, achieving 14.15 ng/mg tissue following buccal administration. **Conclusions**: This work establishes a novel, non-invasive CNS drug delivery platform combining 3D bioprinting with ligand-functionalized ASA NPs to bypass hepatic metabolism and improve brain targeting. The rapid-dissolving ODFs demonstrated high reproducibility, safety, and effective brain deposition, highlighting their translational potential for neurological therapeutics. This approach may be extended to other small molecules with limited CNS penetration, offering a versatile pathway toward precision neuropharmacology.

## 1. Introduction

Buccal administration of small-molecule drugs, delivered either in suspension or encapsulated within nanoparticles, offers a promising, non-invasive strategy for targeting the central nervous system [1,2,3]. The buccal mucosa’s highly vascularized and permeable structure enables rapid drug absorption while circumventing gastrointestinal degradation and hepatic first-pass metabolism [4]. In suspension formulations, drug permeation is primarily controlled by physicochemical properties, where lipophilic molecules predominantly traverse the epithelium via transcellular pathways, and hydrophilic, low-molecular-weight drugs favor paracellular routes [2,3]. Encapsulation of small molecules into nanoparticulate systems, such as dendrimers or polymeric nanoparticles, can enhance stability, protect against enzymatic degradation, and facilitate controlled or sustained release [5]. Additionally, microfold (M) cell-like cells engulf the nanoparticles and transport them across the mucosal epithelium and to mucosal-associated lymphoid tissue (MALT), from which the nanoparticles enter the circulatory system, a process commonly observed in vaccine nanoformulations, thereby facilitating the entry of nanoparticles into the systemic circulation. The anatomical proximity of the facial vein to the internal jugular vein enables efficient drainage of absorbed drugs directly into systemic circulation, further minimizing hepatic metabolism [6]. For drugs particularly susceptible to enzymatic degradation, nanoparticle incorporation offers an added layer of protection, maintaining stability as they circulate to their intended CNS targets [7].

Recent advancements in formulation technologies have further optimized buccal drug delivery systems for CNS applications [8]. Three-dimensional (3D) printing has emerged as a transformative manufacturing tool for personalized buccal films [9,10], offering precise control over drug loading, geometry, thickness, and release kinetics [11,12]. Recent studies promote that printing technologies enhance the patient-centric design of orally disintegrating films, enabling individualized dosing for chronic CNS therapies [9]. Formulation design underscores the role of excipient selection, mucoadhesive polymers, and permeation enhancers in increasing mucosal residence time and optimizing CNS drug delivery [13,14]. Furthermore, the versatility of 3D printing is now widely demonstrated by developing patient-centric buccal films with customizable dosing, adhesion, and controlled release, specifically tailored to meet the unique needs of pediatric, geriatric, and comatose patients by enhancing medication administration and improving therapeutic outcomes in these vulnerable populations [15].

Ischemic stroke is regarded as the second leading cause of death and disability worldwide [16,17]. According to the CDC, in 2022, stroke accounted for 17.5% of all cardiovascular disease deaths [16]. Notably, about 87% of all strokes are ischemic, highlighting their substantial contribution to overall mortality. Beyond the immediate health implications, stroke is a leading cause of long-term disability, significantly reducing mobility in more than half of survivors aged 65 and older [17,18]. This condition is considered to be an acute condition where immediate medical intervention is needed to avoid recurrence of the stroke due to platelet aggregate formation, and also neuroprotectivity as a maintenance strategy [18,19].

ASA, a non-specific COX inhibitor, has been studied extensively for its anti-platelet aggregator (thromboxane A2 inhibitor) [20], thrombolytic activity and neuroprotective properties [21,22,23]. However, its oral administration faces significant challenges in achieving effective brain concentrations due to extensive first-pass hepatic metabolism and the restrictive nature of the blood–brain barrier. Conventional oral or intravenous routes often result in suboptimal CNS bioavailability and increased systemic side effects. Moreover, studies determining the bioavailability of ASA when administered orally are sparse. During ischemic stroke, rapid and targeted delivery of ASA to the brain is critical to minimize neuronal damage [24] and improve clinical outcomes [21,25].

**Figure 1:**
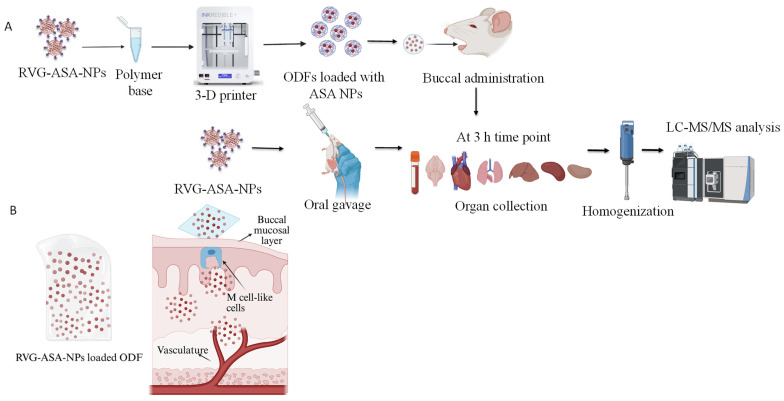
(**A**) Schematics of 3D-printed ASA-BSA NP-loaded oral disintegrating film (ODF) formulation and in vivo evaluation of biodistribution in rat model. RVG-ASA NPs were administered via both oral and buccal routes, while ODFs were administered via the buccal route and biodistribution was assessed using LC-MS/MS. (**B**) Schematic representation of drug absorption following buccal administration of NP-loaded ODFs. Graphics are created using BioRender. Pasu, D. (2025) https://BioRender.com/zz5pyl4.

In this study, we developed 3-D printed oral dissolving films with ASA and its nanoparticles to be administered via the buccal route, as illustrated in Figure 1. The ASA nanoparticles are conjugated with targeting ligands to improve the affinity of the nanoparticles to the brain tissue, thus enhancing the CNS penetration. We explored the formulation of buccal films using a 3-D printer to enable tailored dosing of the brain-targeted ASA nanoparticles and evaluated them for their safety and biocompatibility and determined their biodistribution profiles in both the targeted brain tissue and off-target peripheral tissues, using a murine model.

## 2. Materials and Methods

### 2.1. Materials

Acetylsalicylic acid (ASA), Bovine serum albumin (Fraction V), glutaraldehyde (25% in water) and Acetone were purchased from Fischer Scientific (Pittsburgh, PA, USA). Virus Glycoprotein (RVG) was purchased from GenScript (Piscataway, NJ, USA). Trypsin powder (125 units/mg) was sourced from Fisher Scientific CAS-9002-07-7. Dichloro methane (DCM) was purchased from Sigma-Aldrich (St. Louis, MO, USA). Soluplus® (CAS 936030-92-1) was sourced from BASF (Ludwigshafen, Germany). The chemicals were of analytical grade and used as is without further modification. Dendritic cells (DCs), DC2.4, were received as a kind gift from Dr. Kenneth L. Rock (Dana-Farber Cancer Institute, Inc., Boston, MA, USA). Brain endothelial cells, BEND3 cells, Dulbecco’s Modified Eagle’s Medium (DMEM), fetal bovine serum (FBS), penicillin/streptomycin, and non-essential amino acids were obtained from American Type Culture Collection (ATCC) (Manassas, VA, USA). 8-week-old Swiss Webster mice and CD Sprague-Dawley rats were purchased from Charles River Laboratories (Wilmington, MA, USA). Kollidon^®^ 90F and Kollidon^®^ VA 64 were obtained from BASF (Ludwigshafen, Germany). In vivo studies were conducted in accordance with the approved Mercer University IACUC protocol ref. No. A2310012 and IACUC protocol Ref. No. A2411017.

### 2.2. Formulation of RVG-ASA-NPs Using a 5-Input Chip-Based Microfluidics System

ASA-loaded BSA nanoparticles were prepared using a microfluidic coacervation/nanoprecipitation approach (Dolomite, Herts, UK). An aqueous phase containing ASA and BSA in distilled water was mixed with the organic phase, acetone, at a 1:2 ratio under controlled micromixing, resulting in nanoparticle formation. The suspension was crosslinked with glutaraldehyde and subsequently conjugated with RVG peptide. Trehalose (2%) was added as a cryoprotectant before freezing and lyophilization to obtain dry RVG-ASA-BSA nanoparticles. The final formulation was characterized for its physicochemical properties and yield.

### 2.3. Formulation of ASA Nanoparticles Loaded Oral Dissolving Films Using 3-D Bioprinter

The formulated RVG-ASA-BSA NPs were subsequently loaded onto oral disintegrating films for the buccal route of administration. Various polymers were evaluated for their suitability in fabricating 3D-printed buccal films capable of achieving optimal nanoparticle loading while preserving their physicochemical integrity. Initially, hydroxypropyl methylcellulose (HPMC) was tested as a primary film-forming agent in combination with additives such as citric acid, sucrose, Soluplus^®^ (CAS 936030-92-1, BASF, Ludwigshafen, Germany), and glycerin solution. Additionally, other film-forming agents, including Kollidon^®^ 90F and Kollidon^®^ VA64 (BASF, Ludwigshafen, Germany), were assessed together with PEG 2000 and ethanol, as described in our previous publications [14]. The role of polycaprolactone (PCL), a biodegradable polyester known to enhance the mechanical properties of 3D-printed matrices, was also investigated, as shown in Table 1. Based on physiochemical evaluation, the optimized formulation was prepared by dissolving 4.8 g of Kollidon^®^ 90F (16.24% *w*/*v*) and 0.35 g of Kollidon^®^ VA64 (1.06% *w*/*v*) in 30 mL of ethanol, followed by the addition of 0.18 g of PEG 2000 (0.6% *w*/*v*) into the mixture under continuous stirring for 4 h, with the container covered in foil. This polymer blend, along with ASA-loaded nanoparticles (equivalent to 30 mg ASA per ODF), was then transferred to an 18G nozzle of a CELLINK. INKREDIBLE+^®^ 3D bioprinter (Dolomite, Herts, UK). Diphenyl phosphine oxide (DPPO, 0.1% *w*/*w* relative to total polymer content) was incorporated to generate radicals for UV-assisted matrix stabilization, ensuring mechanical integrity, shape retention, and uniformity without compromising rapid disintegration or biocompatibility [26]. The films were printed out using a CELLINK INKREDIBLE+^®^ 3D bioprinter with a pre-generated G-code file, adjusted for 150 kPa pressure, 6.5 mm/s dispensing speed, 18 G nozzle, and room temperature. The final ODFs were printed directly into custom molds prepared in-lab using the SYLGARD^®^ 186 silicone elastomer kit (10:1 ratio of base to curing agent). After thorough mixing and a 20 min degassing step, the elastomer was used to create custom-made molds to hold the films during printing. Upon printing, the mold with the printed polymer was subjected to UV curing at 405 nm for photo-initiated activation to yield stable films. The plate mold with the films was placed in a heating chamber, set at 55 °C for 10 min and then stored in a desiccator overnight. Blank ODFs were also fabricated following the same procedure, except no nanoparticles were incorporated, and these served as control films. The ODFs were carefully removed from the mold, individually wrapped in aluminum foil pouches, and stored at 25 ± 2 °C with ≤60% relative humidity until further analysis. Blank films were stored under the same conditions as controls. Stability of the films, including appearance, mechanical integrity, and drug content, was monitored over time for physical properties.

### 2.4. Characterization of Oral Dissolving Films Containing ASA Nanoparticles

#### 2.4.1. Morphological Evaluation of the ASA NP-Loaded Oral Dissolving Films

Scanning electron microscopy (SEM) was carried out using a Phenom™ system (Nanoscience Instruments, Phoenix, AZ, USA) to examine the morphology of ODFs incorporated with ASA nanoparticles. To visualize surface morphology, ASA nanoparticle-loaded ODFs were mounted onto metal stubs using double-sided adhesive tape. After being vacuum-coated with a thin layer (100–150 A°) of gold, the nanoparticles were observed under 20 kV using a scanning electron microscope (Phenom World Pure Scanning Electron Microscope, Eindhoven, The Netherlands).

#### 2.4.2. Evaluation of Oral Dissolving Films Containing ASA Nanoparticles Using Fourier Transform Infrared Microscopy (FTIR)

The formulated ODFs, blank, and ASA NP-loaded ODFs were examined using Fourier transform infrared (FTIR) spectroscopy (Shimadzu IRAffinity-1S; Tampa, FL, USA) following previously established methods. Briefly, the blank ODF was placed on the ZnSe crystal platform and analyzed. The same procedure was repeated for the ASA nanoparticle-loaded ODFs, and spectra were measured. Each sample was analyzed three times (*n* = 3), and spectra were recorded. The resulting spectra were compared with each other and with the spectra of individual components to evaluate the drug-polymer interactions in the formulation.

#### 2.4.3. Physicochemical Characterization

The ODFs were characterized for their physicochemical properties, including blank polymer ODFs, blank nanoparticle-loaded ODFs, and ASA nanoparticle-loaded ODFs, to evaluate any significant variations in their structural and functional attributes. The characterization included assessment of thickness and uniformity measurements, mechanical properties such as tensile strength and flexibility, and drug content analysis. Additionally, dissolution studies were performed to evaluate the release profile of ASA from the films.

##### Weight Variation and Diameter

A Metler Toledo electronic balance with a readability of 0.001 mg was used to weigh each formulated film individually. The weight variation was calculated in (mg) for blank polymer ODFs, blank BSA NP-loaded ODFs, and ASA NP-loaded ODFs, each at an *n* = 6 sample size. The average weights and standard deviations were calculated.

##### Diameter

A vernier caliper was utilized to measure the ODFs individually. ODFs from each group (*n* = 6), blank polymer ODFs, blank BSA NP-loaded ODFs and ASA NP-loaded ODFs were measured and the average was recorded.

##### Average Thickness of the Film

A Mitutoyo electronic digital caliper (Mitutoyo Corp., Kawasaki, Japan) was used to measure the thickness of the formulated films. Each individual film from the groups blank polymer ODFs, blank BSA NP-loaded ODFs, and ASA NP-loaded ODFs, each with (*n* = 6), was measured individually. The average thickness in (mm) was reported as mean ± standard deviation (SD).

##### Surface pH of the ODFs

For surface pH determination, each ODF sample was placed in 2 mL of artificial saliva [27,28]. Artificial saliva was prepared using the following process. For 100 mL of artificial saliva, 0.05 g of sodium chloride (NaCl), 0.02 g of potassium chloride (KCl), 0.002 g of calcium chloride (CaCl_2_), 0.002 g of magnesium chloride (MgCl_2_), 0.05 g of sodium bicarbonate (NaHCO_3_), and 0.003 g of sodium phosphate (Na_2_HPO_4_) were dissolved in 100 mL of distilled water in a clean beaker. The mixture was stirred thoroughly to ensure all salts were completely dissolved. After dissolving all the ingredients, the pH of the solution was measured and adjusted to be within the range of 6.5–7.5. Small amounts of sodium hydroxide (NaOH) were added to raise the pH, or hydrochloric acid (HCl) was added to lower it. The pH of the artificial saliva alone was assessed separately by immersing the electrode directly into a beaker containing only the saliva. The pH electrode was carefully positioned on the film’s surface to record the reading. This procedure was repeated for four ODF samples of each formulation, and the resulting pH measurements were used to calculate the mean and standard deviation (SD).

##### Disintegration Test

Since rapid disintegration of the ODFs is beneficial, we tested the disintegration time for each ODF using the optimized formulation. Individual ODF strips were placed in Petri dishes (141 cm^2^), followed by the addition of 3 mL of PBS. The dishes were placed on a shaker to facilitate complete film breakdown. The time taken for each strip to fully dissolve was recorded, with measurements obtained from 4 parallel experiments. The time taken for the ODFs in each group was reported as an average ± SD.

##### Tensile Strength

MARK-10 force gauge (Copiague, NY, USA) was used to measure tensile strength. To provide uniform tension throughout the ODF, the ODF samples were placed between the upper and lower grips of the instrument and firmly attached. The maximum force required at the moment of breakage was noted after the test, which involved exerting force until the film ruptured. After that, the tensile strength was determined and expressed in N/m^2^.

##### Swelling Index

The ODF’s swelling capacity was calculated as per a previously published method [29,30]. The swelling index was evaluated to determine the films’ swelling behavior upon contact with saliva, simulating their application in the oral cavity. The index was calculated by recording the weight of each buccal film before swelling (W1) and after swelling (W2). The films were positioned in Petri dishes on an agar layer and allowed to swell in artificial saliva (pH 6.8) inside a climate-controlled oven, maintained at 37 ± 1 °C. After 180 min, the films were removed from the medium, excess surface water was blotted using filter paper, and the films were reweighed. All measurements were performed in triplicate, and the mean values were reported.

The % swelling index (SI) was calculated using the following formula:

(1) $$\%\;Swelling\;Index\left(SI\right)=\frac{W2-W1}{W1}*100$$

#### 2.4.4. Evaluation of the Anti-Inflammatory Properties of the ASA NPs in ODFs

The ODFs were evaluated for their capacity to encapsulate ASA nanoparticles (NPs) while maintaining the chemical integrity of the drug. The Griess assay, conventionally used to measure nitric oxide (NO) as an inflammatory marker in cells exposed to immunostimulatory agents such as concanavalin A (ConA) [31,32,33,34], was employed here to evaluate the in vitro anti-inflammatory activity of ASA-NPs incorporated into ODFs. A reduction in NO production following treatment with these formulations indicates that ASA retained its biological activity after nanoparticle incorporation and 3D printing. Inflammation was induced in dendritic cells (DCs) using ConA, and the inflamed cells were subsequently treated with ASA–BSA NPs incorporated into ODFs containing 32 µg of ASA for 24 h. Following incubation, NO levels were quantified using the Griess assay. Experimental groups included: cells only (negative control), cells + ConA (positive control), cells + ConA + blank ODFs, and cells + ConA + ASA NP-loaded ODFs.

#### 2.4.5. Determination of Cytotoxicity of the ASA NP-Loaded ODFs Using BEND3 Cells

The cytotoxicity of the ODFs with ASA nanoparticles was evaluated in BEND3 cells (mouse brain epithelial cells) using the MTT (3-(4,5-Dimethylthiazol-2-yl)-2,5-diphenyltetrazolium bromide) assay as per the manufacturer’s instructions, described previously [32,33,34]. Cells were seeded in 96-well plates (1 × 10^4^ cells/well) and incubated overnight for adherence. The ASA ODFs were disintegrated in DMEM, added to the cells in two-fold serial dilutions (500–31.25 µg/mL; *n* = 4 per concentration) and incubated for 24 h at 37 °C. A positive control of cells only and a negative control of DMSO were also added. Following treatment, the medium was replaced with MTT reagent (final concentration 0.5 mg/mL) and incubated for 4 h in the dark. Formazan crystals were dissolved with 200 µL DMSO, and absorbance was measured at 570 nm (background correction at 630 nm) using a BioTek^®^ Synergy H1 microplate reader (BioTek Instruments, Winooski, VT, USA).

#### 2.4.6. Tracking Study Using Indocyanine Green (ICG) Encapsulated BSA Nanoparticles Conjugated with Brain Targeting Ligand RVG, Loaded ODFs to Evaluate the Drug Delivery via Buccal Delivery

A tracking study was performed using the CD Sprague Dawley rat model to monitor RVG-conjugated nanoparticles following buccal administration. The testing was performed as per the approved Mercer University IACUC protocol (animal protocol # A2411017). For this study, a total of 12 rats were utilized, out of which the test group (*n* = 6) received the RVG-ICG NP-loaded ODFs via buccal administration, and the control group (*n* = 6) received RVG-ICG NPs via oral gavage. Indocyanine green (ICG) dye-loaded bovine serum albumin nanoparticles were formulated via nanoprecipitation, followed by glutaraldehyde crosslinking and surface conjugation with brain-targeting RVG ligands, using Microfluidics. These ICG nanoparticles were incorporated into ODFs for buccal delivery. Each ODF contained a dose equivalent to 50 mg/kg of ICG nanoparticles, corresponding to approximately 130–133 mg per film based on the average rat weight of 120–133 g. Prior to administration, rats were anesthetized with an intraperitoneal injection of ketamine (87 mg/kg) and xylazine (13 mg/kg). Anesthesia onset occurred within 10 to 15 min post-injection, lasting 15 to 30 min, followed by a prolonged immobile phase averaging 3.8 h with diminished response to external stimuli. Following anesthesia induction, rats were placed in an isoflurane-infused bioimager to capture real-time imaging of ICG nanoparticle deposition. Images were acquired at multiple time points: 0, 1, 2, 3, 4, 5, 6, 7, 18, and 24 h post-administration.

After imaging, rats were returned to their cages and observed for 5 to 10 min for any signs of respiratory distress or discomfort. Additional monitoring was conducted between 12 and 24 h after doing so to ensure animal welfare. The images acquired from the Bioimager were processed using Li-COR Bioimager Acquisition software V2.4. The fluorescence intensities of the ICG dye localized around the brain region were quantified and compared across the various post-dosing time points.

#### 2.4.7. Assessment of Biodistribution of ASA in Mice Tissues Following Buccal Delivery of Oral Disintegrating Films

Based on the tracking study of brain-targeting nanoparticles following buccal administration, we determined the biodistribution of ASA in the brain and other peripheral tissues at 3 h. The testing was performed as per the approved Mercer University IACUC protocol (animal protocol # A2310012). For this study, we used a murine model of Swiss Webster mice (CFW; Charles River Laboratories, Wilmington, MA, USA) weighing 25–40 g. The mice were housed in groups of four under controlled temperature and humidity, with free access to Laboratory Rodent Diet and water. Two groups, each consisting of *n* = 6 mice, were used for this study. One group was administered by RVG-ASA NP-loaded ODFs, while the other was given RVG-ASA NPs via oral gavage, serving as a control. Each oral dissolvable film (ODF) was loaded with 50 mg/kg of ASA nanoparticles based on mouse body weight, corresponding to approximately 30–33 mg per ODF, as the average mouse weight was ~30–33 g. Prior to ODF administration, mice were anesthetized with isoflurane. During administration, two to three researchers were present: one gently opened the mouse’s mouth using forceps, another placed the ODF into the buccal cavity, and the third monitored and assisted as needed. Mice remained under anesthesia for approximately 10 min to ensure complete dissolution and uptake of the ODF, while being kept warm on a heating mat. Following administration, mice were wrapped in warm blankets and gently stimulated until they regained consciousness. Blood samples were collected via tail snip at 0, 1, and 3 h using 10 µL pipettes. At the 3 h time point (previously determined T_max_), mice were sacrificed, and organs, including the brain, lungs, heart, liver, spleen, kidneys, and blood serum, were harvested and stored using flash-freezing using isopentane. The organs were then analyzed to quantitatively determine the using a dynamic multiple reaction monitoring (dMRM) method using liquid chromatography- triple quadrupole mass spectrometry. Quantitative analysis was performed using an Agilent 5960B HPLC–triple quadrupole mass spectrometer (Agilent, Santa Clara, CA, USA) equipped with an electrospray ionization (ESI) source. Separation was achieved on a ZORBAX RR Eclipse Plus C18 Column, 2.1 × 100 mm, 3.5 µm sourced from Agilent, Santa Clara, CA, USA. The mobile phase consisted of 2% formic acid in a 50:50 (*v*/*v*) acetonitrile–water mixture, delivered isocratically at 0.4 mL/min. Ionization was conducted under positive ESI with standard source settings (350 °C drying gas, 8 L/min flow, 12 psi nebulizer, 3500 V capillary voltage). Dynamic multiple reaction monitoring was performed for the Aspirin 121 ion, in positive polarity. The tissue samples were thawed to room temperature and homogenized using a probe homogenizer and ultracentrifuged. The supernatants were collected and analyzed on the LC-MS/MS with no further dilutions. The distribution of ASA across various organs upon oral and buccal administration of the NPs was quantitatively established from the LC-MS/MS quantification analysis. The results showed significant variation when measured in micrograms per milligram (ng/mg) for tissues and nanograms per microliter (ng/µL) for plasma.

#### 2.4.8. Statistics

All experiments were conducted in triplicate unless indicated otherwise. For statistical analysis, normally distributed data were evaluated using ordinary one-way ANOVA or Brown–Forsythe and Welch ANOVA for independent groups, and two-way ANOVA for dependent groups. Non-normally distributed data were analyzed using the Kruskal–Wallis test. Post hoc comparisons were performed with Tukey’s test for multiple mean comparisons and Dunnett’s test when comparing means to the control group. Mean values ± SEM and corresponding *p*-values were calculated for each experiment using GraphPad Prism version 8.4.3 (GraphPad Software, San Diego, CA, USA).

## 3. Results

### 3.1. Formulation of ASA NP-Loaded with Oral Dissolving Films

All formulations listed in Table 1 successfully produced intact films. Films F1–F4 were HPMC-based, while F5–F8 were Kollidon-based. The oral disintegrating films (ODFs) were evaluated for physical integrity, including mechanical strength, texture, and film-forming ability. Although the HPMC films exhibited desirable film-forming characteristics, physicochemical characterization indicated that the Kollidon-based formulation F5 possessed the optimal combination of mechanical and structural properties and was therefore selected as the preferred formulation.

### 3.2. Physical Characterization of ASA NP-Loaded ODFs

#### Morphological Examination of the ODFs Using Scanning Electron Microscopy (SEM)

**Figure 2:**
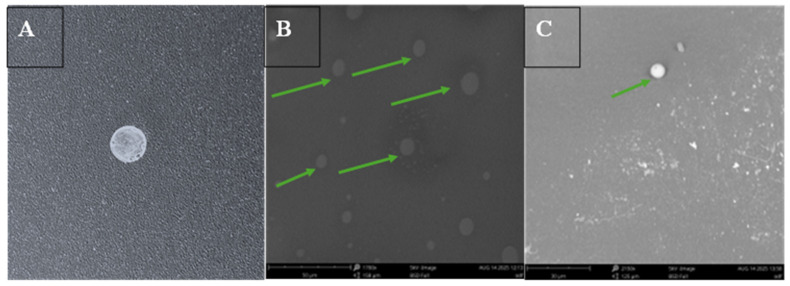
Three-dimensional printed RVG conjugated ASA BSA nanoparticles loaded oral dissolving films (ODF) formulated for buccal administration in mice. (**A**) Digital image of oral dissolving film containing RVG-ASA NPs. (**B**) Scanning electron microscope image at 1700×, 5 kV, BSD full, 50 μm scale of oral dissolving film containing ASA NPs, green arrows representing the presence of nanoparticles in the film. (**C**) Scanning electron microscopy image, 2150×, 5 kV, BSD full, 30 μm to focus on the nanoparticles (represented with a green arrow).

The oral dissolving films were examined for their physical and structural integrity using SEM. The images revealed that the films exhibited a uniform surface with consistent layering and no significant irregularities. ASA nanoparticles embedded within the films maintained their structural integrity, appearing intact with a spherical morphology and showing no evidence of physical damage or aggregation, as shown in Figure 2.

### 3.3. FTIR

**Figure 3:**
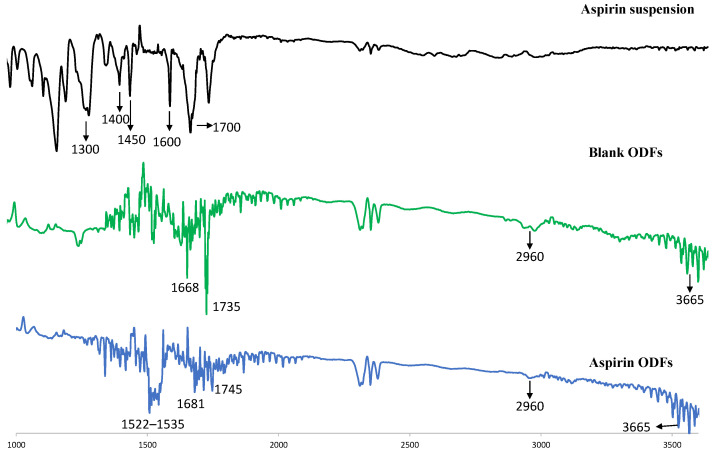
FTIR spectra of formulations. FTIR spectra of (i) ASA suspension, (ii) blank ODF and (iii) RVG-ASA-BSA NP-loaded ODF. All spectra were shown in %transmittance according to the wavelength. Abbreviations: FTIR—Fourier transform infrared spectroscopy, RVG—rabies virus glycoprotein, ODFs—oral disintegrating films.

The incorporation of the ASA nanoparticles in the oral dissolving films was characterized by a Fourier transform infrared (FTIR) spectroscopy method. The FTIR spectra of ASA suspension, blank ODF, and RVG-ASA NP-loaded ODF are shown in Figure 3. The ASA suspension exhibited its characteristic bands at 1300–1450 cm^−1^ (C–O stretching/O–H bending), ~1600 cm^−1^ (aromatic C=C), and ~1700 cm^−1^ (carboxylic C=O). The blank ODF displayed strong polymer-related bands at 1668 cm^−1^ (lactam C=O stretch), 2960 cm^−1^ (C–H stretching), and 3600 cm^−1^ (surface O–H). In the ASA NP-loaded ODF, ASA-specific peaks disappeared, suggesting the absence of crystalline/free ASA. Notably, the carbonyl peak shifted from 1668 cm^−1^ (blank ODF) to 1681 cm^−1^, indicating interaction of the polymer carbonyl with BSA or ASA functional groups. Additionally, a new band at 1522–1535 cm^−1^ appeared, attributed to the amide II vibration of the RVG peptide. 

### 3.4. Physicochemical Evaluation of the Oral Dissolving Films

The formulated 3D-printed oral dissolving films (ODFs) were characterized for average weight (mg), thickness (µm), diameter (mm), disintegration time (min), pH, tensile strength (N/cm^2^), and swelling index. The results are summarized in Table 2. The average weights of the blank polymer-only ODF, blank nanoparticle-loaded ODF, and ASA nanoparticle-loaded ODF were 8.43 ± 0.74 mg, 10.73 ± 0.46 mg, and 10.86 ± 0.46 mg, respectively. The diameters of all three categories were consistent at 0.4 mm, while the average thicknesses were 0.33 mm, 0.47 mm, and 0.47 mm, respectively. Disintegration times measured in artificial saliva averaged 1.38, 2.24, and 2.38 min for the blank polymer ODF, blank nanoparticle-loaded ODF, and ASA nanoparticle-loaded ODF, respectively. All films exhibited an optimal pH range of 7.5–8. Tensile strength measurements showed values of 2.7, 2.6, and 2.7 N/cm^2^ for the blank polymer ODF and 2.3, 2.2, and 2.4 N/cm^2^ for the ASA nanoparticle-loaded ODF, indicating that the incorporation of nanoparticles slightly reduced mechanical strength but maintained sufficient flexibility.

### 3.5. Evaluation of Anti-Inflammatory Properties of the ASA Nanoparticles ODFs

**Figure 4:**
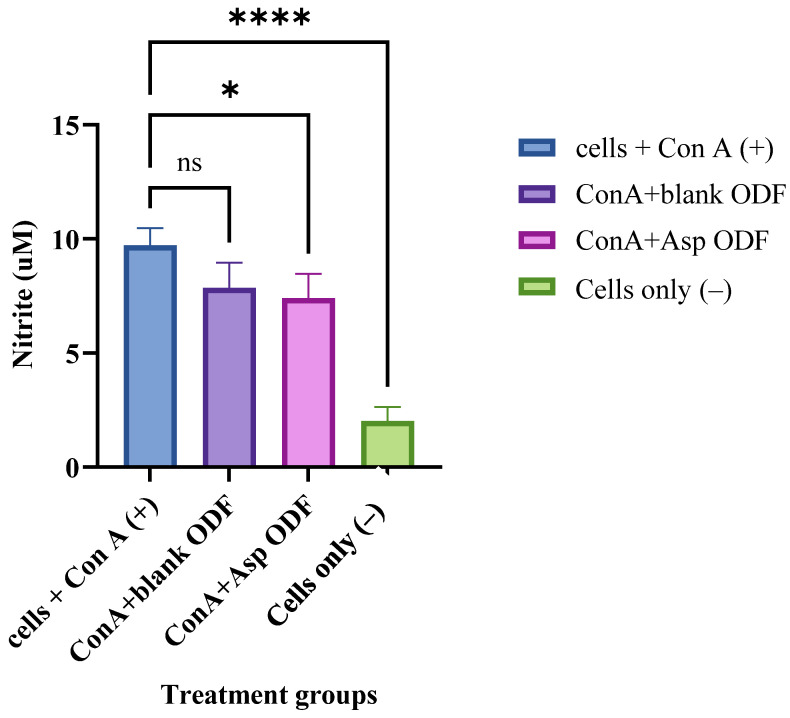
Griess assay measuring nitric oxide (NO) release, expressed as nitrite (µM), from ConA-stimulated dendritic cells. Cells treated with blank ODFs or ASA NP-loaded ODFs were compared. The cell density was adjusted to 1 × 10^4^ cells/well and treated with the following groups for 24 h: concanavalin A (2 µg) (positive control), concanavalin A + blank ODF, concanavalin A + ASA NP-loaded ODF and cells only (negative control). The nitrite released in the supernatant was assessed using Griess’ assay for nitric oxide (NO). Data expressed as mean ±SEM, *n* = 4, one-way ANOVA test, post hoc Dunnett’s multiple comparisons test, ns: non-significant, * *p* ≤ 0.05 and **** *p* ≤ 0.0001.

ASA’s anti-inflammatory activity, mediated through COX inhibition, was evaluated by measuring nitric oxide (NO) levels in nitrite (μM) using a repurposed Griess assay. Experimental groups included cells only, cells + ConA, cells + ConA + blank ODFs, and cells + ConA + ASA nanoparticle (NP)-loaded ODFs. As expected, ConA stimulation significantly increased NO levels compared to untreated cells, confirming successful induction of an inflammatory response (Figure 4). We observed that NO levels decreased following exposure to both blank ODFs and ASA NP–loaded ODFs. The reduction in NO production seen with blank ODFs may be attributed to the intrinsic anti-inflammatory properties of the polymers used in the film matrix. Although the decrease in NO with ASA NP–loaded ODFs was only marginally greater than that observed with blank films, this attenuated response could be influenced by drug release kinetics and potential interactions between the polymer components and the cell culture medium, which may delay the immediate bioavailability of ASA. Overall, the results indicate that the formulated system retained ASA’s anti-inflammatory activity.

### 3.6. Evaluation of the Toxicity of the ASA NP-Loaded ODFs Using MTT Assay

**Figure 5:**
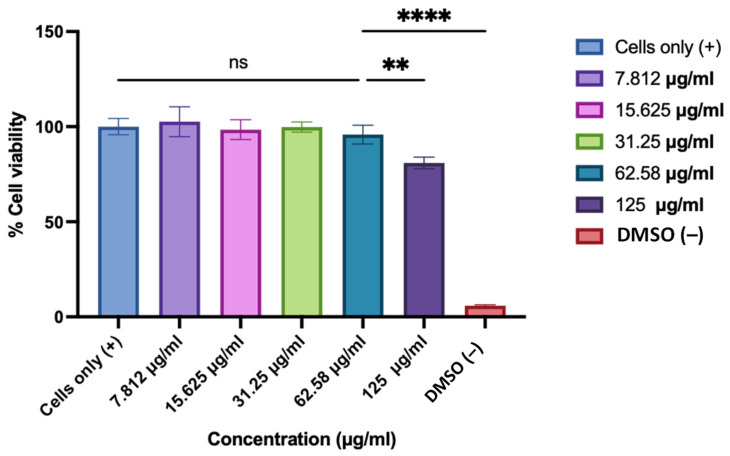
Cytotoxicity of 3D-printed oral dissolving films (ODFs) was evaluated in BEND 3 cells using the MTT assay. The cell density was adjusted to 1 × 10^4^ cells/well. Cells were treated with 2-fold dilutions (concentrations ranging from 125 to 7.8 µg/mL) of ASA NP-loaded ODFs disintegrated in DMEM, with untreated cells as the positive control and DMSO-treated cells as the negative control. Data expressed as mean ± SEM, *n* = 3, one-way ANOVA test, post hoc Dunnett’s multiple comparison test, ns: non-significant, ** *p* ≤ 0.01 and **** *p* ≤ 0.0001.

The cytotoxicity of the 3D-printed oral dissolving films (ODFs) was evaluated using an MTT assay in mouse brain endothelial (BEND 3) cells. Cells were treated with serial 50% dilutions of ODFs disintegrated in DMEM, with untreated cells as the positive control and DMSO-treated cells as the negative control. As shown in Figure 5, the results indicated that ODF concentrations of 62.58 µg/mL and lower did not exhibit significant cytotoxicity compared to the untreated cells, demonstrating an optimal safety profile for the formulated films.

### 3.7. ICG Tracking Study to Evaluate the Nanoparticle Delivery to the Brain Using Bioimager in a Murine Model

**Figure 6:**
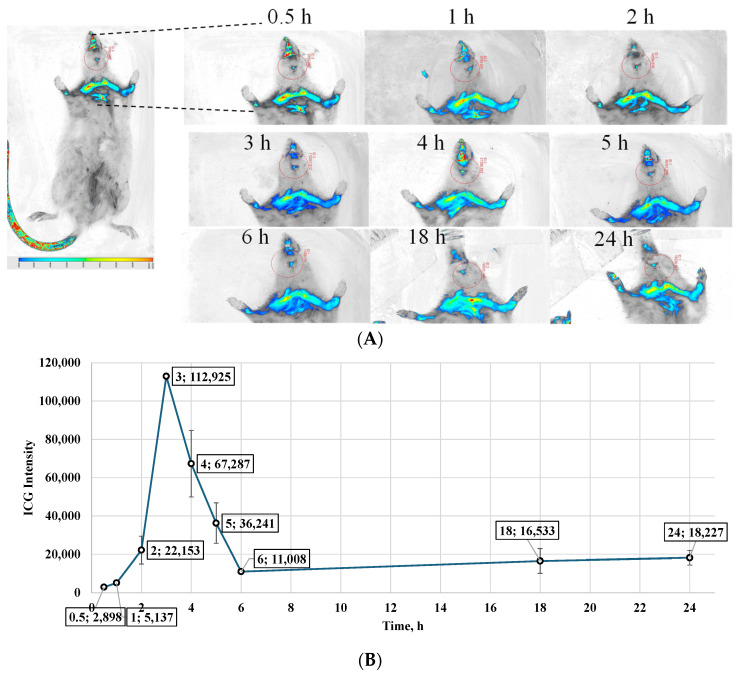
(**A**) In vivo tracking images obtained using a Li-COR Bioimager, depicting ICG fluorescence in the brain region of Sprague Dawley rats after buccal administration of RVG-coated ICG-loaded BSA nanoparticles incorporated into oral dissolving films. Images were captured at multiple time points to monitor nanoparticle transport and deposition. (**B**) Quantitative comparison of ICG fluorescence intensity in the brain region over time, derived from image analysis. The data indicate that maximum deposition occurred at 3 h post-administration, followed by a gradual decline, suggesting clearance from brain tissue. Values are expressed as Mean ± SD (*n* = 6).

A tracking study was performed using brain-targeting indocyanine green (ICG)-nanoparticles incorporated into oral dissolving films (ODFs). Rats were administered by ODFs, and ICG distribution to the brain was monitored at various time points using in vivo bioimaging. The images obtained from the Bioimager were processed using the Li-COR Bioimager software. The intensities of the ICG dye were captured around the brain area and were directly compared among the time points at which the images were obtained after dosing. Quantitative analysis of ICG intensity within the brain region revealed a gradual increase from an average initial value of 4217 at 0 h to a peak of 115,251 at 3 h. The buccal route demonstrated a more sustained drug presence at 6 h, and though the intensity decreased to 191,500, it remained higher than the initial 0 h reading. The buccal route maintained detectable levels of ICG at later time points, with 13,018 at 18 h and 10,405 at 24 h, indicating prolonged retention of the formulation in the brain, compared to the 0 h reading (Figure 6).

### 3.8. Quantitative Determination of the Biodistribution of ASA upon Buccal Administration in a Murine Model

**Figure 7:**
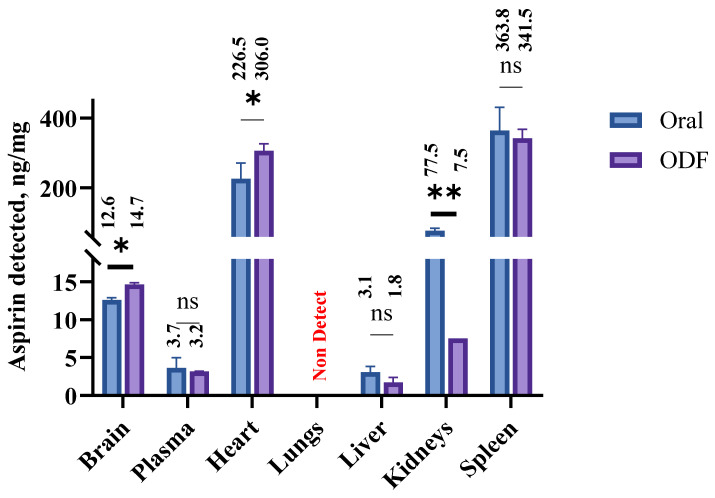
Comparative biodistribution of ASA following oral and buccal administration of ASA nanoparticles incorporated into oral disintegrating films (ODFs) in rats. ASA concentrations in the brain, lungs, heart, liver, spleen, kidney, and plasma were quantified using LC–MS/MS at 3 h post-administration. Data are expressed as mean ± SEM (*n* = 6), statistical comparisons between treatments within each organ were performed using a parametric paired T-test, ns: non-significant, * *p* ≤ 0.05 and ** *p* ≤ 0.01. ASA—acetyl salicylic acid, LC-MS/MS—liquid chromatography-tandem mass spectrometry.

The biodistribution of acetylsalicylic acid (ASA) following oral and buccal administration of brain-targeting ASA nanoparticles incorporated into oral disintegrating films (ODFs) was quantified using LC–MS/MS, and the results are presented in Figure 7. ASA concentrations were analyzed in major organs (brain, lungs, heart, liver, spleen, and kidney) and plasma at 3 h post-administration. After oral administration, the highest concentration of ASA was observed in the spleen (363.75 ng/mg), followed by the heart (176.47 ng/mg) and the kidney (77.45 ng/mg). Moderate levels were detected in the brain (12.6 ng/mg) and liver (3.05 ng/mg), whereas the lungs showed no detectable levels (non-detect). The plasma concentration reached 4.65 ng/µL, indicating systemic absorption.

Following buccal administration via ODFs, a distinct biodistribution profile was observed. The spleen again showed the highest ASA accumulation (341.9 ng/mg), followed by the heart (306.5 ng/mg) and kidney (7.48 ng/mg). The brain concentration increased to 14.15 ng/mg, demonstrating enhanced penetration across the blood–brain barrier (BBB) via the buccal route. The liver exhibited a moderate level (1.75 ng/mg), while no ASA was detected in the lungs (0 ng/mg), similar to the oral route. The plasma concentration was 3.15 ng/µL, confirming systemic exposure. Data are presented as mean ± standard error of the mean (SEM) (*n* = 6).

## 4. Discussion

Encapsulation of bioactive molecules in biodegradable nanoparticles has emerged as an effective strategy to preserve drug stability and bioactivity while minimizing systemic side effects [35,36,37,38,39]. Brain delivery remains challenging due to the restrictive blood–brain barrier and limitations of conventional oral administration, including first-pass metabolism and poor BBB penetration [7,40,41,42]. Ligand-functionalized nanoparticles, such as those coated with RVG, T7, RGD, Angiopep-2, or CAQK, enhance targeting to brain-specific receptors, improving cellular uptake, biocompatibility, and therapeutic precision [35,43,44,45,46]. Buccal administration further facilitates rapid systemic absorption and bypasses first-pass metabolism, offering controlled release and improved patient compliance [3,13,29]. Advanced fabrication techniques, such as 3D printing, enable the creation of automated, precise, reproducible, and patient-tailored formulations [3,9,10,11,14,47,48,49,50,51]. In this study, RVG-coated ASA nanoparticles were embedded in 3D-printed oral dissolving films, integrating targeting, buccal delivery, and advanced manufacturing to maximize brain delivery and minimize systemic exposure [35,45], thus providing a promising approach for acute ischemic stroke therapy. Unlike the several methods reported so far for formulating ODFs, such as solvent casting [13,22], 3D printing eliminates gaps in formulation accuracy and precision and reduces the need for personnel [47]. In this study, we explored eight formulations, including four HPMC-based films and four Kollidon-based films. The HPMC formulations included glycerin, citric acid, sucrose, and Soluplus for their plasticizer, pH-adjusting, and sweetening properties. Kollidon formulations utilized Kollidon 90F and Kollidon VA64 polymers, along with PEG 2000, as the plasticizer. Although the HPMC films exhibited optimal physical characteristics such as plasticity, flexibility, and texture, their disintegration time was 7–10 min, which is not ideal for rapid-release ODFs, similar to what was previously reported [13,52]. In contrast, the Kollidon films, specifically Formulation F5, exhibited the best characteristics among the tested oral dissolving films. The thickness of F5 was uniform and within the desirable range (~50–100 µm), ensuring consistent drug content while maintaining patient acceptability and ease of handling. The tensile strength and folding endurance indicated that F5 had sufficient mechanical robustness to withstand handling and storage without cracking or breaking, while still retaining the flexibility needed for buccal administration. Notably, the disintegration time was less than 3 min, which is rapid compared to HPMC-based formulations, highlighting its suitability for fast-release buccal delivery. While the disintegration test was performed in phosphate buffer, testing in simulated saliva could provide additional physiological relevance. Future studies could include simulated saliva to better mimic in vivo oral conditions. SEM imaging confirmed uniform nanoparticle incorporation, and Future studies will include quantitative content uniformity assessments to validate consistent drug deposition. FTIR analysis confirmed successful ASA encapsulation, RVG conjugation, and polymer compatibility without chemical degradation. Functional assays, including the Griess and MTT tests, verified that ASA NP-loaded films maintained chemical integrity, effective release, and cellular safety.

To evaluate the brain-targeting potential and pharmacokinetics of the nanoparticle-loaded ODFs upon buccal administration, RVG-conjugated ICG-labeled BSA nanoparticles were incorporated into 3D-printed buccal films and tracked in a rat model using a bioimager. The findings suggest that the buccal films disintegrated effectively, facilitating the rapid release of nanoparticles into systemic circulation and enabling subsequent transport to the brain. The observed distribution profile also provided valuable insights into the temporal kinetics of brain-targeted delivery via this route, indicating both an initial burst phase within the initial 3 h and a prolonged presence of nanoparticles.

The biodistribution analysis of acetylsalicylic acid (ASA) following oral and buccal administration of RVG-ASA-BSA nanoparticles incorporated into ODFs revealed route-dependent distribution patterns consistent with the pharmacokinetic characteristics of these delivery pathways. conventional oral administration is often associated with delayed onset, extensive first-pass metabolism, and significantly reduced drug penetration into the central nervous system due to the restrictive nature of the blood–brain barrier (BBB) [53,54,55,56]. In this study, both oral and buccal routes were tested using RVG-conjugated nanoparticles, enabling direct assessment of the impact of the absorption pathway on CNS delivery. The buccal route bypasses first-pass metabolism, allowing more rapid systemic availability of the nanoparticles [3,11,23,51,57,58,59], as demonstrated by the early brain localization observed in this study. The brain uptake observed here can be attributed to the RVG (rabies virus glycoprotein) conjugation, which facilitates receptor-mediated transport across the BBB via nicotinic acetylcholine receptors expressed on brain endothelial cells [35,41,45,60,61]. From a clinical perspective, such a delivery strategy could offer significant advantages in the management of acute ischemic stroke, where rapid drug availability in the brain is critical to limiting neuronal damage and improving therapeutic outcomes [62,63,64,65,66].

ASA concentrations detected in the brain after both administration routes were comparable and numerically close, yet the difference was statistically significant. The detection of ASA in brain tissue at a concentration of 14.15 ng/mg following buccal administration is particularly noteworthy, demonstrating the route’s ability to enhance the systemic availability of the NPs, achieving approximately 0.9% of the administered dose within the brain. Similarly, oral administration resulted in a brain concentration of 12.6 ng/mg, corresponding to about 0.75% of the administered dose. In comparison, a previously published study [67] involving oral suspension administration in mice reported ASA levels of 60 µg/g (equivalent to 60 ng/mg) in brain tissue, corresponding to approximately 0.4% of the administered dose. These findings indicate measurable penetration across the blood–brain barrier (BBB), which is a critical consideration for effective central nervous system (CNS) drug delivery. The overall enhancement in the drug delivery with both routes can be attributed to nanoparticle encapsulation and RVG conjugation strategies. Since both routes used RVG-functionalized nanoparticles, the slightly higher CNS delivery observed with buccal administration likely reflects its absorption pathway, which bypasses hepatic metabolism and enables effective systemic availability of intact nanoparticles. 

Although we anticipated that the two administration routes would produce more pronounced differences in tissue distribution, the overall biodistribution profiles were largely similar. In both administration routes, the spleen and heart demonstrated pronounced ASA accumulation, reflecting preferential uptake in highly perfused and immunologically active tissues. This distribution is consistent with the known bio-disposition of albumin-based nanoparticles, which are readily internalized by splenic macrophages and can accumulate in vascularized tissues [68,69,70]. The elevated cardiac levels are likely due to the biodistribution properties of the nanoparticles and the heart’s rich vascularization, which facilitates their uptake. However, the higher cardiac concentrations observed with oral administration may also reflect rapid systemic peaks of free ASA generated during gastrointestinal absorption and partial nanoparticle degradation. In contrast, buccal administration moderated peripheral exposure and reduced nanoparticle degradation, while still maintaining effective CNS targeting. The absence of ASA in the lungs may reflect minimal pulmonary recirculation following buccal absorption and rapid systemic distribution [69,70]. Although the precise therapeutic threshold of ASA in the brain during ischemic stroke is not clinically established, preclinical studies in rodent models have shown that oral administration of ASA at 30 mg/kg significantly reduced infarct volume, improved neurological outcomes, and inhibited platelet aggregation [67,71]. Given that our buccal formulation was administered at 50 mg/kg, the observed brain concentration of 14.15 ng/mg suggests a pharmacologically meaningful exposure, potentially exceeding the effective range reported in previous studies. The RVG-conjugated BSA nanoparticles were engineered to ~150 nm, facilitating mucosal absorption and efficient transport across the BBB. Furthermore, surface functionalization with the RVG peptide enabled receptor-mediated targeting to the CNS, increasing the fraction of drug reaching the brain compared to non-targeted formulations. These properties, along with the buccal delivery route, likely contributed to the improved brain distribution that was observed. Clinically, these findings highlight the translational promise of buccal and RVG-functionalized nanoparticle delivery systems to achieve rapid and targeted CNS exposure. This is an advantage particularly valuable in acute ischemic stroke, especially in critical settings where early intervention is often hindered by delayed absorption or patient unresponsiveness due to neurological deficits. This strategy holds promise for managing acute neurological conditions such as ischemic stroke, offering rapid onset, improved compliance, and enhanced therapeutic precision.

## 5. Conclusions

This study demonstrates that 3D-printed buccal oral dissolving films (ODFs) incorporating RVG-functionalized nanoparticles provide a rapid, reproducible, and customizable platform for targeted CNS drug delivery. Technology enables precise control over drug loading, uniform film quality, and on-demand fabrication, highlighting its potential for personalized medicine in neurological disorders. By combining faster onset, brain-targeted delivery, and sustained exposure, these buccal ODFs equipped with RVG-ASA NPs represent a potential alternative to conventional oral administration for central nervous system therapeutics.

Future studies will evaluate ASA delivery in pathological models, in vivo, such as ischemic stroke or traumatic brain injury, to determine how BBB disruption may influence drug deposition and efficacy. These investigations will further clarify the therapeutic potential of RVG-ASA NPs ODFs for acute and chronic neurological conditions.

## Figures and Tables

**Table 1:** Formulation combinations of various polymers in preparation of oral dissolving films containing ASA nanoparticles using CELLLINK INCREDIBLE 3-D printer.

Ingredients	F1	F2	F3	F4	F5	F6	F7	F8
Kollidon 90F (g)					4.9	3	4.9	-
Kollidon VA64 (g)					0.32	1	-	0.32
PEG 2000 (g)					0.18	1	0.18	0.18
Ethanol (mL)					30	30	30	30
PCL (g)					-	3	0.3	4.9
Glycerin (50%) mL	5	-	-	-				
citric acid (g)	1	1	0.25	0.25				
HPMC ^1^ (g)	2	2	2	2				
Sucrose (mL)	0.5	0.5	0.1	0.1				
Soluplus (mL)	0.5	0.5	0.1	0.1				
Water (mL)	80	30	20	60				

^1^ HPMC = hydroxypropyl methyl cellulose; PCL = polycaprolactone.

**Table 2:** Physiochemical evaluation of the 3-D printed oral disintegrating films.

S. No.	Parameter	Blank Polymer ODFs	Blank NP Loaded ODFs	ASA NP Loaded ODFs
1	Weight variation (mg)	8.43 ± 0.74	10.73 ± 0.46	10.86 ± 0.28
2	Diameter (mm)	0.4 ± 0.03	0.4 ± 0.01	0.4 ± 0.01
3	Thickness (mm)	0.33 ± 0.10	0.47 ± 0.24	0.47 ± 0.26
4	Disintegration Test (min)	1.38 ± 0.29	2.24 ± 0.24	2.38 ± 0.28
5	pH	7.5	8	7.5
6	Tensile Strength (N/cm^2^)	2.67 ± 0.06	2.28 ± 0.5	2.30 ± 0.10
7	Swelling Index (%)	48 ± 0.8	56 ± 0.7	63 ± 0.3

## Data Availability

The original contributions presented in this study are included in the article. Further inquiries can be directed to the corresponding author.
